# Impact of withholding early parenteral nutrition completing enteral nutrition in pediatric critically ill patients (PEPaNIC trial): study protocol for a randomized controlled trial

**DOI:** 10.1186/s13063-015-0728-8

**Published:** 2015-05-01

**Authors:** Tom Fivez, Dorian Kerklaan, Sascha Verbruggen, Ilse Vanhorebeek, Sören Verstraete, Dick Tibboel, Gonzalo Garcia Guerra, Pieter J Wouters, Ari Joffe, Koen Joosten, Dieter Mesotten, Greet Van den Berghe

**Affiliations:** Clinical Department and Laboratory of Intensive Care Medicine, Academic Division Cellular and Molecular Medicine, KU Leuven University and Hospital, Herestraat 49, B-3000 Leuven, Belgium; Intensive Care Unit, Department of Pediatrics and Pediatric Surgery, Erasmus Medical Center, Sophia Children’s Hospital, Rotterdam, The Netherlands; Department of Pediatrics, Intensive Care Unit, University Alberta, Stollery Children’s Hospital, Edmonton, AB Canada

**Keywords:** Critical illness, Children, Nutrition, Sepsis, Lipid, Protein, Glucose, Infection

## Abstract

**Background:**

The state-of-the-art nutrition used for critically ill children is based essentially on expert opinion and extrapolations from adult studies or on studies in non-critically ill children. In critically ill adults, withholding parenteral nutrition (PN) during the first week in ICU improved outcome, as compared with early supplementation of insufficient enteral nutrition (EN) with PN. We hypothesized that withholding PN in children early during critical illness reduces the incidence of new infections and accelerates recovery.

**Methods/Design:**

The Pediatric Early versus Late Parenteral Nutrition in Intensive Care Unit (PEPaNIC) study is an investigator-initiated, international, multicenter, randomized controlled trial (RCT) in three tertiary referral pediatric intensive care units (PICUs) in three countries on two continents. This study compares early versus late initiation of PN when EN fails to reach preset caloric targets in critically ill children. In the early-PN (control, standard of care) group, PN comprising glucose, lipids and amino acids is administered within the first days to reach the caloric target. In the late-PN (intervention) group, PN completing EN is only initiated beyond PICU-day 7, when EN fails. For both study groups, an early EN protocol is applied and micronutrients are administered intravenously. The primary assessor-blinded outcome measures are the incidence of new infections during PICU-stay and the duration of intensive care dependency. The sample size (n = 1,440, 720 per arm) was determined in order to detect a 5% absolute reduction in PICU infections, with at least 80% 1-tailed power (70% 2-tailed) and an alpha error rate of 5%. Based on the actual incidence of new PICU infections in the control group, the required sample size was confirmed at the time of an *a priori-* planned interim-analysis focusing on the incidence of new infections in the control group only.

**Discussion:**

Clinical evidence in favor of early administration of PN in critically ill children is currently lacking, despite potential benefit but also known side effects. This large international RCT will help physicians to gain more insight in the clinical effects of omitting PN during the first week of critical illness in children.

**Trial registration:**

ClinicalTrials.gov: NCT01536275 on 16 February 2012.

## Background

### Nutritional support for children in intensive care

The state-of-the-art nutrition used for critically ill children is essentially based on expert opinion, small studies with surrogate endpoints and extrapolations from adult studies or from studies in healthy children outside the ICU. It is widely accepted that in healthy children, nutrition not only serves to maintain body tissues but also allows growth, which is considered of particular importance during infancy and adolescence [1,2]. In hospitalized children, especially in the young, the current European and American guidelines for nutrition recommend early parenteral nutrition (PN) to prevent/correct malnutrition and to sustain appropriate growth when enteral nutrient (EN) supply is insufficient [3,4]. Observational studies suggest that about a quarter of children, most notably infants, admitted to pediatric intensive care units (PICUs) develop a pronounced caloric deficit [1]. The stores of energy, fat and protein in children are limited, leaving children to rely on muscle mass to provide necessary substrates for metabolism. The energy deficit observed with acute critical illness in children has been associated with adverse outcome [5]. Based hereon, it is current practice in PICUs to start PN in the acute phase of critical illness to supplement insufficient EN with the intention to avoid underfeeding [3,4]. However, overfeeding may also be harmful [6-9]. It is difficult to administer the correct amount of nutrition, avoiding overfeeding as well as underfeeding.

### Varying nutritional guidelines and clinical practices

It is currently advised to assess energy expenditure considered to reflect energy requirements, through the use of indirect calorimetry during the course of critical illness and to use this technique for determining individualized targets to guide nutritional therapy [10]. However, a European survey conducted in 2004 showed that only 17% of the PICUs use this technique [11] and the technique itself has not been well standardized [12,13]. In the most critically ill, major caveats are present, such as respiratory support with more than 40% oxygen and the use of uncuffed tubes resulting in unpredictable measures. The use of standard equations to predict energy expenditure and/or requirements also carries the risk of overfeeding and underfeeding [14-16].

Experts worldwide agree that there are insufficient data to make evidence-based recommendations for the optimal target of caloric intake in critically ill children and for the optimal time after onset of critical illness by which this target should be reached. The lack of widely accepted caloric targets for critically ill children results in nutritional strategies that vary substantially across centers. The current European and American guidelines for nutrition in hospitalized children recommend PN to prevent or correct malnutrition and to sustain appropriate growth when EN supply is insufficient [10,17]. Most guidelines advise doing this early so that the recommended daily allowances for children are reached on day 2 or 3 after PICU admission. These recommendations are based on evidence from cohort studies without a control group, case series or expert opinion (Grade D level).

The ongoing controversy on optimal amount, composition and timing of administration of PN in critically ill children may in fact conceal the fact that there is no hard evidence for any use of PN in critically ill children. Supported by the results of a Cochrane systematic review, Joffe *et al*. concluded that randomized trials investigating the role of intravenous nutritional support during the first week of critical illness in children should be performed and should include a control arm in which no nutritional support is administered or hypocaloric goals (below basal metabolic rate) for nutritional support are used [18].

### Rationale of the study and study hypothesis

A recent randomized controlled trial (RCT) in critically ill adults [19] showed that the early provision of PN worsened rather than improved outcomes as compared with withholding PN and thus tolerating a substantial caloric deficit up to 1 week in ICU. Also, other studies did not show clinical benefit of early PN in adult ICU patients [20,21]. Hitherto, no well-designed RCT has been performed in critically ill children. The aim of the PEPaNIC trial (the acronym stands for Pediatric version of the effect of Early Parenteral Nutrition to complete insufficient enteral nutrition in ICU patients) is to investigate whether a strategy of withholding PN during the first 7 days in the PICU (late PN) provides clinical benefit over the current practice of early PN in critically ill children. We hypothesize that withholding PN for 1 week in the PICU reduces new infections and shortens the duration of PICU-stay.

This hypothesis is currently being tested in a multicenter superiority RCT performed in three large, tertiary referral PICUs (University Hospitals Leuven, Leuven, Belgium; Erasmus Medical Center, Sophia Children’s Hospital, Rotterdam, The Netherlands; Stollery Children’s Hospital, Edmonton, AB, Canada). The centers were invited to participate based on a self-declared routine use of early PN in the PICU. It was anticipated that this routine use of early PN differs among centers. This was considered to be an asset as it contributes to the external validity of the PEPaNIC trial.

## Methods/Design

### Ethical approval

The study protocol and (deferred) informed consent forms were approved by the institutional ethical review boards in Leuven, Belgium (ML8052 Amend-ID0005), Rotterdam, The Netherlands (NL38772.000.12) and Edmonton, AB, Canada (Pro00038098). Informed consent is given in writing by the parents or the legal guardians, confirmed by the child when older than 7 years, after providing all information orally in plain language and in writing. For planned admissions, informed consent is obtained prior to surgery/procedure. For unplanned admissions, informed consent is obtained within 24 hours after admission on the PICU (deferred informed consent as the nutritional therapy should be initiated from PICU admission onward).

### Patients’ eligibility - Inclusion criteria

Upon admission to the participating PICUs, all critically ill children are screened for nutritional risk and eligibility for inclusion in the PEPaNIC clinical study [22]. All non-eligible patients, identified by the local investigators, are logged.

Critically ill children, newborn to 17 years (inclusive or exclusive depending on the local definition of a pediatric patient) old, with a STRONGkids (Nutritional risk score) score of 2 points or more and who are likely to stay in the PICU for more than 24 hours, are eligible for inclusion [22].

### Exclusion criteria

Patients fulfilling one or more of the following criteria are excluded:

- STRONGkids score lower than 2 on PICU admission [22]
- Not critically ill (for example, anticipated oral intake within 24 hours)
- Non-pediatric patients (aged 17 or older, compare with above)
- Premature newborns (<37 weeks gestational age upon admission in the PICU)
- ‘Do not resuscitate’ code at the time of PICU admission
- Expected death within 12 hours
- Readmission to PICU after already having been randomized
- Enrollment in another intervention trial
- Transfer from another PICU or neonatal ICU after a stay of more than 7 days
- Ketoacidotic or hyperosmolar coma
- Inborn metabolic diseases requiring specific diet
- Short bowel syndrome or other conditions requiring PN for more than 7 days prior to PICU admission

### Data collection at study entry

At baseline, data on demographic (age, gender, race/ethnicity, (pre-)admission bodyweight and height) and clinical characteristics of the patients are obtained. For all patients, severity of illness scores are calculated such as the PEdiatric Logistic Organ Dysfunction PELOD score and, for cardiac surgery patients, the Risk-Adjustment in Congenital Heart Surgery or RACHS score. The Pediatric RISk of Mortality (PRISM) score cannot be used for this study as the nutritional management is expected to affect the highest blood glucose concentration during the first 24 hours. In addition, co-morbidities prior to admission are noted. These comprise, among others, the presence of a genetic syndrome, gestational age at birth, presence/history of cancer, diabetes mellitus, kidney failure and infection upon admission.

### Randomized treatment allocation

#### Randomization procedure

Randomization to early PN or late PN in a 1:1 ratio, is performed centrally (KU Leuven, Belgium) by use of a dedicated computerized system, accessible in all centers around the clock, 7 days a week. The computer algorithm allocates every consecutive, eligible patient per center to one of the two treatment arms in a blinded fashion by use of permuted blocks per diagnostic stratum to create parallel groups. The block size is unknown to bedside physicians, nurses and members of the research team. Patients are stratified per study site according to age groups (<1 year and ≥ 1 year) and the following primary diagnostic categories on admission:

I. Medical-PICU admissions (infectious or non-infectious): (a) neurological (b) other.
II. Surgical-PICU admissions (elective or emergency) according to referral discipline (a) cardiac surgery (b) other.

#### Treatment allocation and blinding

Concealed allocation to the randomized treatment was realized by use of the computerized randomization system described above. It was considered not feasible to blind treating physicians and patients for the allocated treatment during the time window of the randomized intervention. After discharge to the normal ward, all treating physicians are unaware of the randomized treatment allocation. All outcome assessors and investigators not directly involved in the patients care, such as statisticians, infectious disease specialists and laboratory personnel, are fully blinded to treatment allocation.

#### Common strategy for early EN in both study arms

The initiation and increase of EN, and the use of gastroprokinetics are prescribed in the standing orders for EN in each center. Both groups receive micronutrients (trace elements, minerals and vitamins) intravenously from day 2 onwards until the amount of EN given reaches 80% of the caloric target.

#### Randomized interventions

Patients randomized to the early-PN strategy (standard of care or control group) receive this type of nutrition according to current management in each of the participating centers, which were recruited based on a routine use of early PN. For patients randomized to the late-PN group (intervention group), all PN is withheld during the first week in the PICU. The international setting of the trial brings some variation in the control group (see study rationale and hypothesis), while the intervention group is strictly standardized (‘no PN during the first week in PICU’).

#### Standard of care or control group: early-PN

*In the Leuven* (*BE*) *PICU*, patients randomized to the early-PN group receive a mixture of glucose 30% and Vaminolact® (Fresenius, Uppsala, Sweden) in equal amounts upon admission to PICU, comprising 150 mg/ml glucose and 4.7 mg/ml nitrogen. For patients who require fluid restriction, total fluid intake is 50 ml/m^2^/h on days 1 and 2 (the day after admission and further referred to as day 2), and 60 ml/m^2^/h on day 3. Patients not requiring fluid restriction receive 100 ml/kg/day for the first 10 kg bodyweight, 50 ml/kg for the next 10 kg, and 20 ml/kg for the bodyweight over 20 kg, to be reached within 3 days. For all patients on intravenous (IV) nutrition, and within the fluid limitation described above, lipids (SMOFlipid® (20 g/100 ml), Fresenius, Uppsala, Sweden) are added from the second morning after admission, initially at a dose of 1.5 g/kg/day, increasing to a maximum of 3 g/kg/day, depending on the age. On the third morning after admission, pharmacy-prepared PN preparations are prescribed, unless adequate enteral nutritional intake is expected. PN preparations contain a mixture of glucose 50% and SMOFlipid® covering respectively 60 to 70% and 40 to 30% of calculated energy target and a 1.5 to 2.5 g/kg protein intake, according to age, by Vaminolact®. If the body weight is above 5 kg, Vaminolact® is replaced by Vamin 18® (Fresenius, Uppsala, Sweden). Any enterally-delivered energy is taken into account twice daily to reduce the energy delivered by PN. When EN covers 80% of optimal calculated caloric needs, PN is stopped. When the patient starts to take oral nutrition, the PN and/or EN is reduced and eventually stopped. Whenever enteral or oral intake falls below 50% of calculated caloric needs, the PN is restarted.

*In the Rotterdam* (*NL*) *PICU*, patients randomized to the ‘early-PN’ group receive a continuous glucose infusion upon admission to PICU (<30 kg; 4 to 6 mg/kg/min, > 30 kg; 2 to 4 mg/kg/min). From day 2 onwards the glucose intake is increased for all children on IV nutrition to 8.3 mg/kg/min (5 to 10 kg), 6.9 mg/kg/min (10 to 30 kg) or 4 mg/kg/min (>30 kg). Primene® (Baxter, Kobaltweg 49, 3542 CE Utrecht) (5.5 to 5.7 mg/ml nitrogen) is added from day 2 onward at 25 ml/kg/day (<10 kg) or 20 ml/kg/day (10 to 30 kg). From day 2 onwards, Intralipid® (Baxter, Kobaltweg 49, 3542 CE Utrecht) is added initially at a dose of 10 ml/kg/day (<10 kg) or 7.5 ml/kg/day (10 to 30 kg), increasing to 20 or 15 ml/kg/day respectively. For patients who require fluid restriction, intake is adjusted accordingly. Children > 30 kg on IV nutrition receive from day 2 onwards Olimel N5 (Baxter, 5.2 mg/ml nitrogen, 115 mg/ml glucose) when central lines are in place or Olimel N4 (Baxter, 4.0 mg/ml nitrogen, 75 mg/ml glucose) when only peripheral lines are in place; the dose is 48 ml/kg/day. Any enterally-delivered energy is assessed twice daily and the energy delivered by PN is reduced accordingly. Energy goals for enteral nutrition are based on the body weight-based Schofield equation [23] (first day of admission) and on the Recommended Dietary Allowances (RDA, Dutch Health Council) for the subsequent length of stay (Dietary Reference Intake: energy, protein and digestible carbohydrates, 2001, Health Council of the Netherlands: The Hague). Energy goals and composition of parenteral nutrition are based on the European Society of Pediatric Gastroenterology, Hepatology and Nutrition (ESPGHAN) guidelines [4]. When EN covers 80% of calculated caloric needs, PN is stopped. When the patient starts with oral nutrition, PN and/or EN is reduced and eventually stopped. Whenever enteral or oral intake falls below 50% of calculated caloric needs, PN is restarted.

*In the Edmonton* (*CA*) *PICU,* the patient’s energy expenditure is assessed upon admission by a registered dietitian when possible. Nutritional support is initiated as soon as possible, with the goal to match energy expenditure (measured or estimated resting energy expenditure of the child). The urgency of initiation of nutrition support is dependent on nutritional risk prior to admission, disease state and age. If indirect calorimetry cannot be done, 65% of basal metabolic rate is used Food and Agriculture Organization-World Health Organization (FAO-WHO) to determine caloric requirement. This number is adjusted daily by the dietitian based on the acute phase response and clinical picture of the child. If nutritional requirements cannot be met enterally, PN is added to achieve caloric target. On admission to PICU, patients receive a glucose infusion of approximately 3 to 4 mg/kg/minute taking into account the total fluid prescribed by medical staff. At that time EN is initiated when possible. On the morning of day 2, if the patient is not already on full enteral feeding, 20% IV lipids are initiated at 0.5 g/kg/day. On the morning of day 3, if the patient is not already on full enteral feeding, lipid infusion is increased to 1 g/kg/day and a solution of amino acids and concentrated glucose is added. The caloric goal is Basal Metabolic Rate when the patient is intubated and Total Energy Expenditure when the patient has been extubated.

#### Intervention group: late-PN

In the 3 centers, patients randomized to the late-PN group receive a mixture of glucose 5% and NaCl 0.9% at, respectively, 60% and 40% of the total flow rate that is required to obtain optimal hydration, as prescribed by the attending physician, taking into account the volume of EN that is being delivered. No other forms of PN (lipid or protein infusions) are administered. When the amount of EN that is administered still covers less than 80% of the calculated targets after 1 week in the PICU, supplemental PN is initiated on day 8 according to the current PN protocols in each center.

The medical and nursing staff of the PICU were all informed and trained extensively during regular meetings before the start of the trial and were familiarized with the protocol. In order to optimize protocol compliance, the protocol was programmed in the patient data management system (PDMS). The use of this program was explained to every nurse, trainee and resident on the PICU and was always supervised by the senior staff.

Adherence to the protocol in Leuven and Rotterdam was guaranteed by using a PDMS guided system and by careful follow-up by study nurses. In Edmonton, a paper protocol was used and adherence checked by an independent study nurse and physician.

#### Criteria for stopping the study intervention

When in the intervention arm (late-PN group), blood glucose concentration falls spontaneously (without exogenous insulin) below 50 mg/dl, the standard infusion of glucose 5% is switched to 10% glucose until blood glucose concentration is higher than 80 mg/dl and stable. Thereafter, the infusion of glucose 10% is stopped again and switched back to glucose 5%.

### Blood glucose management

In Leuven, patients in both study groups receive continuous insulin infusion to target blood glucose levels of 50 to 80 mg/dl when aged < 1 year and 70 to 100 mg/dl when aged ≥ 1 year. Blood glucose and potassium are monitored systematically every 1 to 4 hours on the blood gas analyzer (ABL Radiometer, Copenhagen, Denmark) using undiluted arterial blood samples drawn via a VAMP® system (Edwards Lifescience Pontbeekstraat 4 1702 Groot-Bijgaarden), Edwards Lifescience Pontbeekstraat 4 1702 Groot-Bijgaarden [24] and insulin infusion is adjusted when needed.

In Rotterdam, patients in all age groups receive continuous insulin infusion using a step-wise nurse-driven glucose control protocol to target blood glucose levels of 72 to 145 mg/dl, except for patients with traumatic brain injury for whom the target is set at 108 to 145 mg/dl [25]*.* Blood glucose and potassium are monitored systematically every 1 to 3 hours on the blood gas analyzer (ABL 625; Radiometer, Copenhagen, Denmark) using arterial or capillary blood samples.

In Edmonton, patients in all age groups receive continuous insulin infusion at the discretion of the attending physician when blood glucose levels exceed 180 mg/dl. The attending physician sets the lower target range limit.

### Other procedures and guidelines

Other medical treatments are not described by the study protocol. Patients are weaned from the ventilator and from hemodynamic support according to standardized guidelines used in each participating PICU. End-of-life decisions, when further intensive care is considered to be futile, are taken in consensus by senior PICU physicians and the referring specialist.

### Handling of re-admissions to the PICU

Patients who are readmitted to the PICU after a participation in PEPaNIC are not eligible for reinclusion. Patients who are readmitted to the PICU within 48 hours of discharge and who are still within the 7 days’ time window of the initial randomization receive the nutrition strategy they were randomly assigned to during the initial PICU admission. Patients readmitted more than 48 hours after PICU discharge will be fed at the discretion of the attending physician (standard care).

### Outcome measures

#### Primary endpoints

The two primary endpoints of this RCT are: (i) the incidence of new infections during PICU- stay and (ii) the duration of PICU dependency. The latter will be reported as the crude number of PICU-stay days and as the time to live discharge from PICU, to account for mortality as a competing risk.

Also, the proportion of patients from the intention-to-treat population who stayed 8 days or more in PICU will be reported. This is not only reflecting the proportion of prolonged critically ill patients but also examines effects of the randomized intervention beyond the time window of the randomized intervention in PICU.

The incidence of new infections for all patients in the three centers will be scored in consensus by the same two assessors (infectious disease specialists), who are blinded for treatment allocation. This assessment is based on an *a priori*-drafted protocol [19], which makes use of prescribed antibiotics and clinical infection and inflammation data.

As the timing of PICU discharge to a regular ward may be affected by the availability of beds on regular wards, which could induce bias, we *a priori* decided to analyze ‘time to discharge from PICU’ as ‘time to *ready for* discharge from PICU’. A patient is considered ‘ready for discharge’ as soon as all clinical conditions for PICU discharge have been fulfilled (no longer in need for, or at risk of, vital organ support).

#### Secondary safety endpoints

Secondary safety endpoints comprise: (i) death during PICU-stay and during the time window of the randomized intervention (up to day 8), (ii) the proportion of patients with at least 1 episode of severe hypoglycemia (<40 mg/dl), (iii) in-hospital mortality and (iv) 90-day mortality. As a specific Serious Adverse Event (SAE), hypoglycemia resistant to bolus administration of glucose during the time window of the randomized intervention will be reported for both groups.

#### Secondary efficacy endpoints

1. *Time to* (*live*) *discharge from hospital and duration of hospital stay*, for both the index hospitalization and total hospitalization including stay in the referred hospital.
2. *Time to final* (*live*) *weaning* from mechanical respiratory support and duration of mechanical ventilation.
3. *Kidney failure*. Proportion of patients in need for renal replacement therapy (RRT) during PICU-stay and the duration of RRT (for those patients requiring RRT). Also, the further analysis of the maximum and daily serum level of creatinine and urea during the intervention window and during PICU-stay will be reported. Other plasma and urine markers of kidney function will be investigated.
4. *Need for pharmacological or mechanical hemodynamic support* during PICU-stay and duration of such need. In addition, time to final (live) weaning from all pharmacological or mechanical hemodynamic support in PICU will be analyzed.
5. *Number of readmissions to the PICU*. The proportion of patients readmitted within 48 hours after discharge will be recorded. Also the proportion of patients readmitted to the PICU beyond 48 hours during their index hospital stay will be reported, as these patients will have been excluded from treatment allocation and will receive standard care.
6. *Liver dysfunction*. Markers of liver function will be measured and proportion of patients with abnormal tests will be compared.
7. *Inflammation*. Effect of the intervention on inflammation will be analyzed by comparing markers of inflammation. Both peak values and time courses will be analyzed.
8. *Duration of antibiotic treatment*. The duration of antibiotic treatment (whenever given) within the intervention window and during the PICU-stay will be compared between the groups.
9. *Nutrition delivered during PICU-stay.* The macronutrients and calories administered during the intervention window and thereafter during PICU-stay will be compared between the treatment groups. Total amount of macronutrients, as well as the amounts administered parenterally and enterally, will be reported.
10. *Structural and functional differences in muscle tissue during PICU-stay*. By ultrasonography, skeletal muscle thickness of the quadriceps, as a marker of muscle wasting, will be reported in a subset of patients. In addition, handgrip strength will be measured in a subset of patients older than 6 years.
11. *Intolerance to enteral feeding during PICU-stay.* Markers of tolerance to enteral feeding will be determined in a subset of patients. Markers in blood, stool and buccal swab samples will be investigated.

Further pre-planned studies (execution depending on further funding), of which the detailed protocols and the methods for statistical analysis will be reported separately, are here listed below:

1. *Direct healthcare-related costs*. Total, direct healthcare costs during index PICU-stay will be compared between the treatment groups [26].
2. *Mechanistic studies*. Explanations of any observed effects of delayed administration of PN as compared with standard of care will be assessed. These will comprise, among others, metabolic, endocrine, inflammation and (epi)genetic analyses, the investigation of the role of severity of illness, the use of indirect calorimetry, the type of blood glucose management, and post-randomization factors such as type and dose of administered macronutrients, and disease evolution [27].
3. *Long-term follow-up*. This will include developmental and neurocognitive assessments, metabolic, endocrine, inflammation and (epi)genetic studies, with a healthy matched control group investigated over time in parallel.

### Data collection following recruitment

All systemically applied medications received by the patients during the stay in PICU are registered. Every day the quantities of kilocalories, carbohydrates, lipids and proteins delivered by either PN or EN are calculated and entered into the electronic Case Record Form (eCRF). The need for and the number of days of mechanical ventilatory support, of mechanical and pharmacological hemodynamic support, of renal replacement therapies, days on antibiotics and days requiring a central line are recorded. Blood, urine, buccal mucosal swabs and hair samples are taken upon PICU admission and during PICU-stay. Such samples are appropriately handled (collected on ice when required) and immediately stored (at room temperature or at −20°C/−80°C as appropriate) for future measurements. Analyses on blood and urine for the primary clinical analyses include routine chemistry, hematology, and markers of inflammation. Further metabolic, endocrine, inflammatory and (epi)genetic measurements on stored samples in the context of mechanistic analyses are planned. For mechanistic and exploratory studies, ultrasound evaluation of the skeletal muscle, in combination with muscle strength measurements will be performed in a subset of patients [28-30]. Quality of life on admission and after 4 to 6 months is recorded through a validated, semi-structured questionnaire, filled out by the parents, which is repeated at 2 and 4 years after enrollment in the PEPaNIC trial.

### Data handling and record keeping

Data are collected electronically in an anonymized eCRF, unambiguously linked to the source file. Data are manually transferred and checked for accuracy into the eCRF by the clinical research assistants’ team on a daily basis. Extensive range and consistency checks are performed by the study monitor. All original records, such as consent forms, eCRFs and relevant correspondence, will be archived at the participating centers, according to the local regulations. Vital status at 90 days (and at later follow-up times) will be recorded for all patients, by the National Death Registries. When this information is not available, vital status will be checked through the hospital information system or the regional network of pediatricians and general practitioners.

All data are stored anonymously. Investigators involved in the trial do not have direct access to the database. In addition, the study monitor has logged the use of the database. After the trial, the study monitor will store all data in a secured file that is only accessible by the study monitor himself.

### Trial organization

The sponsor (KU Leuven) provides direct access to the eCRF, the source data and the study master file for monitoring, for review by the independent ethics committee and regulatory inspection. The sponsor established an independent data safety monitoring board (DSMB). The sponsor appointed one monitor. The monitor verifies that the trial is performed in accordance to the protocol as described in the European Medicine Agency’s ‘Note for guidance on good clinical practice CPMP/ICH/135/95.’ as well as the *Declaration of Helsinki*. Monitoring is performed and reported according to the sponsor’s standard operating procedures. The clinical research team guarantees a daily follow-up of patient screening and inclusion, availability of requested clinical data in the clinical patient files and protocol compliance. Non-compliance to the protocol and other questions or problems are reported to the study monitor and discussed with the principal investigators and trial steering committee. SAEs are reported to the study sponsor and, if needed, to the local ethics committee. The study monitor regularly provides the sponsor and the DSMB with reports on inclusions and SAEs. Regular meetings are organized with principal investigators and clinical research teams to discuss the daily progression of the PEPaNIC trial.

The protocol has been instructed in each hospital to all clinical medical and nursing staff through frequent teaching sessions and clinical feedback rounds. The protocol decision support is integrated into the PICU PDMS in Leuven and Rotterdam, facilitating the prescription of the exact amounts of PN and EN according to protocol and clinical evolution.

In order to achieve adequate participant enrollment to reach target sample size, regular meetings and site visits take place every 3 months together with the Rotterdam team and via teleconferences with the Edmonton team.

Regular data auditing is done by the administrative trial team, the DSMB and by the central independent audit procedure in place at the University Hospital of Leuven in compliance with the European Trials Directives.

### Statistical analysis plan

One Consolidated Standards of Reporting Trials (CONSORT) diagram will be reported.

*Protocol compliance* will be documented by comparing the actual amounts of PN and EN during the intervention window and this will be reported as absolute numbers of calories and weight units.

For the primary and secondary endpoints taking place during PICU-stay all data will be available. In case of request for discontinuation of the study intervention by patients, parents or legal guardians, this will be respected, but all data will be analyzed. In case of consent withdrawal, the parents will be asked whether the data can be used for analysis. In case this would not be allowed, all data of that patient will be removed from the database, and this will be reported in the CONSORT diagram. At all time, the intention-to-treat principle will be respected and reported. No data imputation will be undertaken for any of the primary or secondary outcomes.

*Variables* will be summarized as frequencies and percentages, means and standard errors of the means, or medians and interquartile ranges, as appropriate.

*Results* will be analyzed with the use of chi-square testing, Student’s *t*-test or non-parametric testing (Wilcoxon rank-sum test, Van der Waerden test or Median test), as appropriate. Kaplan-Meier plots will be used to document time-to-event effects, and the time-to-event effect size will be estimated with the use of Cox proportional-hazard analysis. All time-to-event analyses will also be performed on data censored at 90 days. As death is a competing risk for duration of care outcomes, non-survivors will be censored beyond the longest duration of such care required for survivors [19]. All outcomes will be analyzed both with and without adjustment for baseline risk factors, including the diagnostic and age groups, severity of illness, severity of nutritional risk and center. The latter is considered necessary to account for the differences among centers in nutrition given to the control group and the variation in blood glucose control targets. For these analyses, *P*-values will be considered significant when at or below 0.05 without correction for multiple comparisons. To assess whether any eventual impact of the intervention on the primary endpoints is affected by the baseline risk factor subgroups, interaction *P*-values will be calculated (logistic regression or Cox proportional hazard analysis) with a threshold for significance of interaction set at a *P*-value of < 0.1. All analyses will be conducted on an intention-to-treat basis.

### Sample size calculation and interim analyses

In the design phase of the PEPaNIC trial, and based on the previous adult EPaNIC trial results, the sample size (N = 1,440, 720 patients per arm) was determined in order to detect a reduction in the incidence of new infections during PICU-stay from 20 to 15% (Absolute Risk Reduction 5%), with at least 80% 1-tailed power and at least 70% 2-tailed power and at an alpha error of 5%. With this sample size, the trial can also detect a major safety issue, such as a doubling of the PICU mortality rate from 4% (the baseline mortality in the Leuven center) to 8% with a statistical power of 89% in a 2-sided test with an alpha error of 5%. This sample size will also allow to detect a reduction in mean duration of stay in PICU of 1 day with at least 90% power (2-tailed) and 95% certainty.

Two *interim analyses* of the safety endpoints (except 90-day mortality) only were planned (after inclusion of 480 upon specific request of the DSMB, and after inclusion of 50% of the study population). It was *a priori* decided to determine the actual incidence of new infections during PICU-stay in the 3 centers, as this was not known exactly for each of the participating centers prior to trial initiation. In order to allow statistical repowering and to judge the necessity of inclusion of more trial sites, the assessment of incidence of new infections during PICU-stay in the control group took place after inclusion of 750 patients. Based on this actual incidence of new PICU infections in the control group, the hypothesized absolute risk reduction of 5% and an alpha error rate of 5%, the sample size of 1,440 patients (720 patients in each arm) was found sufficiently large to yield a statistical power of 77% 2-sided and of 85% 1-sided. As these interim analyses did not assess any of the efficacy endpoints, no adjustments of the *P*-values are needed.

## Discussion

The clinical evidence for the administration of PN in critically ill children is missing [18]. Thousands of children are annually exposed to this non-evidence-based treatment, which is assumed to result in faster recovery (benefit). This large international RCT will help PICU physicians to obtain more insight into the possibility of the omission of PN during the first week of critical illness. A significant difference in the safety and/or efficacy endpoints will provide important evidence for optimizing clinical patient care. Also a neutral result will provide important insight, as this would mean that clinicians can safely withhold PN in all comparable patients during the first week of ICU stay, which would have an impact on healthcare spending in the PICU.

## Trial status

The study was initiated as planned on 18 June 2012. At the time of the safety interim analyses (after 480 and 750 study patients discharged from PICU), the DSMB advised the continuation of the trial and ratified the initial sample size of 1,440 patients as adequate to test the hypothesis. On 1 December 2014, 1,130 patients have been included into the PEPaNIC trial. Recruitment of the last patient is expected for October 2015.

## Abbreviations

CONSORT: Consolidated Standards of Reporting Trials
DSMB: data safety monitoring board
eCRF: electronic case record form
EN: enteral nutrition
ESPGHAN: European Society of Paediatric Gastroenterology, Hepatology and Nutrition
FAO-WHO: Food and Agriculture Organization-World Health Organization
PDMS: patient data management system
PELOD: PEdiatric Logistic Organ Dysfunction
PEPaNIC: impact of withholding early parenteral nutrition completing enteral nutrition in pediatric critically ill patients - Pediatric Early versus Late Parenteral Nutrition in Intensive Care Unit
PICU: pediatric intensive care units
PN: parenteral nutrition
PRISM: Pediatric RISk of Mortality
RACHS score: Risk-Adjustment in Congenital Heart Surgery score
RCT: randomized controlled trial
RDA: Recommended Dietary Allowances
RRT: renal replacement therapy
SAE: serious adverse event
